# Scaling an Evidence-Based Program: The Case of the Tailored Activity Program

**DOI:** 10.1093/geroni/igaa057.2159

**Published:** 2020-12-16

**Authors:** Sokha Koeuth, Katherine Marx, Laura Gitlin, Catherine Piersol

**Affiliations:** 1 Drexel University, Philadelphia, Pennsylvania, United States; 2 Johns Hopkins University, Baltimore, Maryland, United States; 3 Thomas Jefferson University, Ardmore, Pennsylvania, United States

## Abstract

The Tailored Activity Program (TAP) is a proven program delivered primarily by occupational therapists addressing dementia-related clinical symptoms including caregiver well-being. Although used in 9 countries including the United States, scaling and widespread dissemination is challenging. We discuss key revisions to TAP to facilitate dissemination including matching assessments to those used in different practice settings, translation of materials into different languages, providing worksheets to help trainees adapt TAP to local contexts and a training/certification online experience using story board, an interactive media integrated onto the Blackboard learn management system, to provide on-demand training modules. The learning platform allows learners to engage with others, preview modules and share experiences. Revisions enable greater flexibility for program adaptation yet adherence to its core principles. With over 150 trainees, we use REAIM to evaluate effectiveness of modifications and to understand implications for its reach. Part of a symposium sponsored by the Behavioral Interventions for Older Adults Interest Group.

